# Supplementary material for: Association between oral microbiome and seven types of cancers in East Asian population: a two-sample Mendelian randomization analysis
Source: Front Mol Biosci. 2023 Nov 21;10:1327893. doi: 10.3389/fmolb.2023.1327893 (PMC10702768; doi:10.3389/fmolb.2023.1327893)
Supplement: Supplementary file 1 [file DataSheet1.ZIP › Supplementary Materials/MR plots for tongue/tongue═╝/Lung cancer/pheno.3007_to_lung cancer_leave_one_out.pdf]

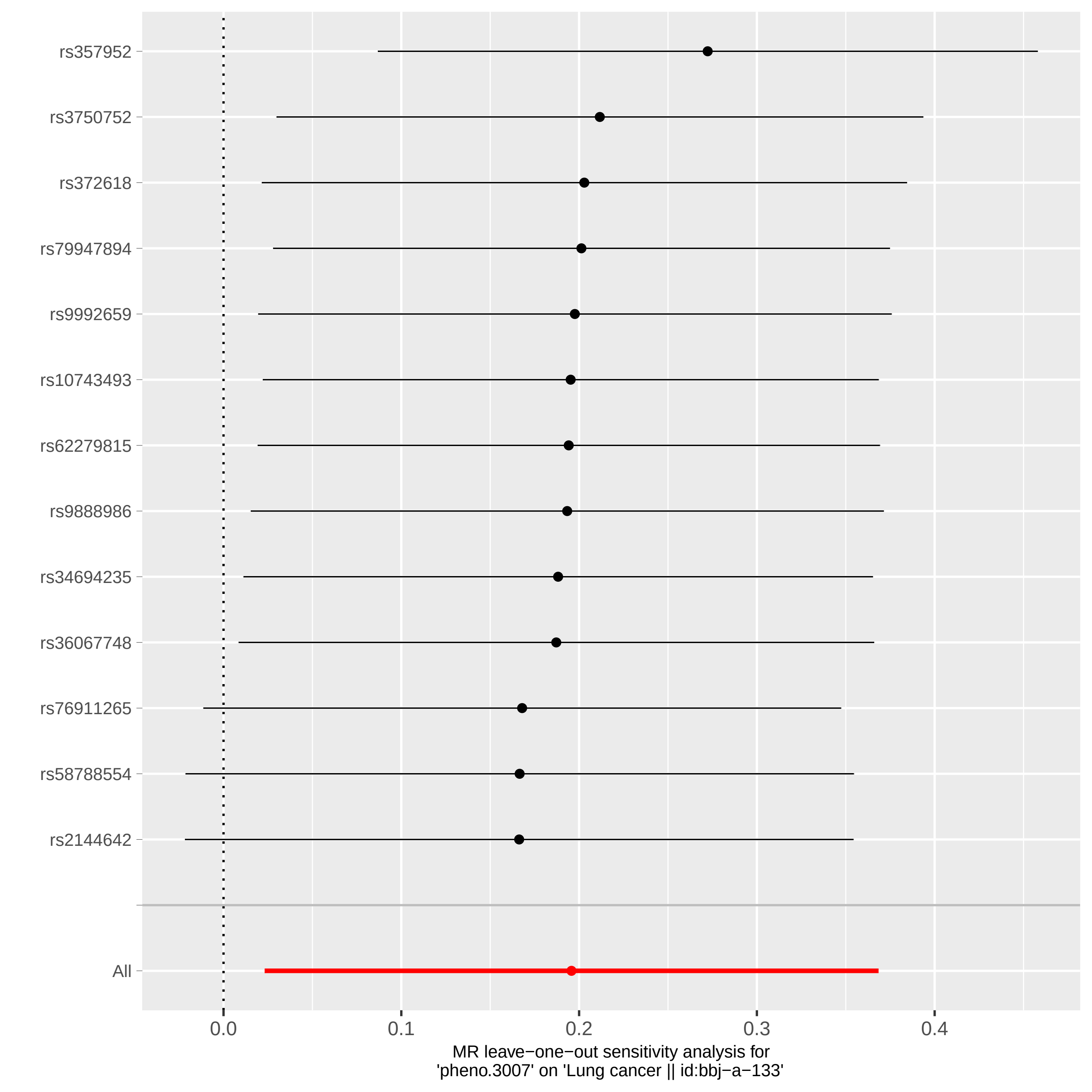
rs357952
rs3750752
rs372618
rs79947894
rs9992659
rs10743493
rs62279815
rs9888986
rs34694235
rs36067748
rs76911265
rs58788554
rs2144642
All
0.0
0.1
0.2
0.3
0.4
MR leave−one−out sensitivity analysis for
'pheno.3007' on 'Lung cancer || id:bbj−a−133'
